# Supplementary material for: Repeat Variants, Biomarkers, and Molecular Signatures in Parkinson’s Disease: ATXN2, ATXN3, CACNA1A, PRNP, TBP, C9ORF72, TOMM40, APOE, and POLG—A Swedish Perspective
Source: Int J Mol Sci. 2025 Sep 20;26(18):9213. doi: 10.3390/ijms26189213 (PMC12470772; doi:10.3390/ijms26189213)
Supplement: Supplementary file 1 [file ijms-26-09213-s001.zip › 3. ijms-3811078 Supplementary tables & figures.pdf]

Repeat Variants, Biomarkers, and Molecular Signatures in Parkinson's Disease: *ATXN2*, *ATXN3*, *CACNA1A*, *PRNP*, *TBP*, *C9ORF72*, *TOMM40*, *APOE*, and *POLG*—A Swedish Perspective

Jose Miguel Laffita-Mesa 1,2,*, Martin Paucar 1 and Per Svenningsson 1,*

1: Department of Clinical Neuroscience (CNS), K8, Neuro Svenningsson, BioClinicum J5:20, Karolinska Institutet, 171 64 SOLNA Stockholm, Sweden.

2: Department of Neurobiology, Care Sciences and Society, BioClinicum, Akademiska Stråket 1, J10:20, Karolinska Institutet, 171 64 SOLNA Stockholm, Sweden.

Correspondence to: PhD Jose Miguel Laffita-Mesa, Professor Per Svenningsson,
Department of Clinical Neuroscience, Karolinska Institutet, 17176 Stockholm, Sweden
E-mails: jose.laffita@ki.se, per.svenningsson@ki.se

Table S2. Full cohort description.

Attribute	Categories	% (N=161)
**Apoe Haplotype**	E2	1.9
	E3	65.2
	E4	32.9
**TOMM40 Genotype**	L_L	1.86
	L_VL	13.04
	S_L	16.15
	S_S	22.36
	S_VL	31.68
	VL_VL	14.91
**Summed TOMM40 Genotype**	Intermediate	70.19
	Long	29.81
**POLG Genotype**	10/11Q_10/11Q	82.6
	Non-10/11Q_10/11Q	16.8
	Non-10/11Q_Non-10/11Q	0.6
**Gender**	Male	67.08
	Female	32.92
**Ethnicity**	Sweden	81.99
	Caucasian	2.48
	Iran	1.86
	Other (e.g., Finland, France)	0.62 each
**Phenotype**	PD	99.38
	PD + Dementia	0.62
**Ever Smoked**	No	65.22
	Yes	34.78
**Exposed to Pesticides**	No	84.47
	Yes	7.45
	UK	8.07
**Heredity PD**	No	65.84
	Yes	34.16

Table S3. Clinical parameters of the PD cohort (N=161).

Attributes [references values]	Mean	Std. Dev	Max	Min	CI95%	Number of values
**Age at diagnoses**	62.95	10.55	89.0	36.00	61.27-to-64.63	154
**Disease Duration**	4.97	5.47	23.00	0.00	4.0-to-5.94	125
**Caffeine habits cups per day**	2.80	2.05	10.00	0.00	2.44-to-3.16	127
**MDS-UPDRS-I**	2.97	1.99	10.00	0.00	2.62-to-3.32	126
**MDS-UPDRS-II**	10.44	5.72	27.00	1.00	9.44-to-11.45	126
**MDS-UPDRS-III**	25.19	11.75	57.00	7.00	23.09-to-27.28	123
**MDS-UPDRS-IV**	3.97	2.66	17.00	0.00	3.49-to-4.44	124
**MDS-UPDRS tot**	42.34	17.94	91.00	12.00	39.13-to-45.56	122
**Hoehn and Yahr**	2.50	1.07	5.00	1.00	2.33-to-2.67	156
**Schwab and England [100%]**	82.94	17.86	100.00	20.00	79.4-to-86.48	100
**MADRS-S**	9.18	7.81	33.00	0.00	7.72-to-10.64	112
**HADS A [21]**	5.65	4.24	18.00	0.00	4.84-to-6.47	107
**HADS D [21]**	4.39	3.59	16.00	0.00	3.71-to-5.08	107
**HADS totalt [42]**	10.15	7.43	32.00	0.00	8.74-to-11.56	109
**MMSE (MMT) [30]**	26.48	3.78	30.00	18.00	25.36-to-27.6	46
**MoCA [30]**	23.42	4.63	30.00	8.5	22.61-to-24.23	128
**β-Amyloid [>450 ng/L]**	944.2	321.7	1780.00	252.00	863.8-to-1025.0	64
**fosfo-Tau [<60 ng/L]**	41.18	17.29	110.00	16.00	36.9-to-45.47	65
**Tau [<300 ng/L]**	224.5	147.5	1090.00	60.00	188.0-to-261.1	65

Table S4. Detailed Regression Results Table.

Parameter estimates	Variable	Estimate	Standard error	95% CI (asymptotic)	|t|	P value	P value summary
β0	Intercept	66.82	12.73	40.88 to 92.76	5.25	<0.00	****
**β1**	**REPEATS**	**7.93**	**4.14**	**-0.50 to 16.37**	**1.92**	**0.06**	**ns**
β2	APOE E4	2.59	3.28	-4.10 to 9.27	0.79	0.44	ns
β3	TOMM40	0.33	3.26	-6.31 to 6.97	0.10	0.92	ns
β4	POLG	2.65	4.04	-5.58 to 10.87	0.66	0.52	ns
β5	MALE?	-1.41	3.44	-8.43 to 5.61	0.41	0.68	ns
**β6**	**DD**	**-0.81**	**0.42**	**-1.67 to 0.05**	**1.93**	**0.06**	**ns**
β7	Heredity	-2.50	2.94	-8.49 to 3.48	0.85	0.40	ns
β8	UPDRS	0.07	0.13	-0.21 to 0.33	0.46	0.65	ns
β9	H&Y	3.51	2.35	-1.28 to 8.29	1.49	0.15	ns
β10	MADRS	-0.38	0.33	-1.04 to 0.28	1.17	0.25	ns
β11	MoCA	-0.25	0.40	-1.06 to 0.57	0.61	0.54	ns
β12	β-Amyloid	-0.01	0.01	-0.02 to 0.009	0.79	0.44	ns
β13	fosfo-Tau	-0.36	0.24	-0.85 to 0.13	1.49	0.15	ns
**β14**	**TAU**	**0.08**	**0.033**	**0.013 to 0.15**	**2.45**	**0.02**	*

Table S5. Analysis of Variance.

Regression	SS	DF	MS	F (DFn, DFd)	P value
**Variables**	2320	14	165.7	F (14. 32) = 2.07	P = 0.044
**REPEATS**	**293.9**	**1**	**293.9**	**F (1. 32) = 3.67**	**P = 0.06**
APOE E4	49.68	1	49.68	F (1. 32) = 0.62	P = 0.44
TOMM40	0.8257	1	0.86	F (1. 32) = 0.01	P = 0.92
POLG	34.42	1	34.42	F (1. 32) = 0.43	P = 0.52
MALE?	13.43	1	13.43	F (1. 32) = 0.17	P = 0.69
**DD**	**297.5**	**1**	**297.5**	**F (1. 32) = 3.72**	**P = 0.06**
Heredity	58.11	1	58.11	F (1. 32) = 0.73	P = 0.40
UPDRS	17.24	1	17.24	F (1. 32) = 0.21	P = 0.65
H&Y	178.7	1	178.7	F (1. 32) = 2.23	P = 0.15
MADRS	109.8	1	109.8	F (1. 32) = 1.37	P = 0.25
MoCA	30.21	1	30.21	F (1. 32) = 0.38	P = 0.54
β-Amyloid	49.44	1	49.44	F (1. 32) = 0.62	P = 0.44
fosfo-Tau	176.7	1	176.7	F (1. 32) = 2.21	P = 0.15
**TAU**	**479**	**1**	**479**	**F (1. 32) = 5.98**	**P = 0.02**
Residual	2563	32	80.09		
Total	4883	46			

Table S6. Considerations for genetic aggregation in Parkinson's disease (PD) in our cohort.

1. **Family History and Clustering**
• **Multiple relatives affected by PD within the same family.**
• **Affected relatives across at least two generations strengthen the evidence for aggregation.**
2. Degree of Relatedness
• **Closer relatives carry a higher weight in establishing genetic aggregation:**
• **First-degree relatives (parents, siblings, children): Strongest indication of aggregation.**
• **Second-degree relatives (grandparents, aunts, uncles, half-siblings): Moderate indication.**
• **Third-degree relatives (cousins, great-grandparents): Weaker indication but may still contribute when combined with other evidence.**
3. Number of Affected Relatives
• **Two or more first-degree relatives affected by PD (with or without other neurodegenerative conditions) significantly suggest aggregation.**
• **If both parents are affected, consider potential genetic inheritance patterns.**
4. Age of Onset
• **Early-onset PD (<50 years): Stronger association with genetic aggregation.**
• **Late-onset PD: More likely influenced by multifactorial (genetic and environmental) factors but does not rule out aggregation.**
5. Molecular Evidence
• **Presence of pathogenic variants in known PD-related genes (e.g., LRRK2, PARK2, SNCA, GBA).**
• **Risk polymorphisms in common variants (e.g., MAPT, GBA).**
6. Phenotypic Consistency
• **Relatives share a similar clinical presentation or atypical features associated with known genetic forms of PD.**
7. Population and Ethnic Considerations
• **Certain populations have higher prevalence of genetic forms (e.g., LRRK2 G2019S in Ashkenazi Jews, North African Berbers, GBA NORTBOTTIAN).**
Example Threshold for Aggregation
• **At least one first-degree relative with PD or related disorders (e.g., dementia, atypical parkinsonism) is often enough to consider genetic aggregation in clinical or research settings.**

Table S7. Search strings in pubmed and EMBASE for the discussion.

("Parkinson Disease"[MeSH Terms] OR "Parkinson's Disease"[Title/Abstract]) AND ("Genetics"[Subheading] OR "Genetic Predisposition to Disease"[MeSH Terms] OR "Genes"[MeSH Terms]) AND ("Sweden"[MeSH Terms] OR "Sweden"[Title/Abstract]).
("Parkinson Disease"[MeSH Terms] OR "Parkinson's Disease"[Title/Abstract]) AND ("Genetic Variation"[MeSH Terms] OR "Genome, Human"[MeSH Terms]) AND ("Sweden"[MeSH Terms] OR "Sweden"[Title/Abstract])
('parkinson disease'/exp OR parkinson) AND disease:ti,ab AND ('genetics'/exp OR 'genetic predisposition to disease'/exp OR 'genes'/exp) AND ('sweden'/exp OR sweden:ti,ab)
("Parkinson Disease"[MeSH] OR "Parkinsonism"[MeSH] OR "Parkinson's Disease"[Title/Abstract] OR "Parkinsonism"[Title/Abstract]) AND ("C9ORF72"[MeSH Terms] OR "C9ORF72"[Title/Abstract] OR "C9ORF72 protein, human"[MeSH Terms])
(("ATXN3"[MeSH Terms] OR "Ataxin-3"[MeSH Terms]) OR ("PRNP"[MeSH Terms] OR "Prion Proteins"[MeSH Terms])) OR ("CACNA1A"[MeSH Terms] OR "Calcium Channels, P/Q-Type"[MeSH Terms]) AND ("Parkinson Disease"[MeSH Terms] OR "Parkinsonism"[MeSH Terms])

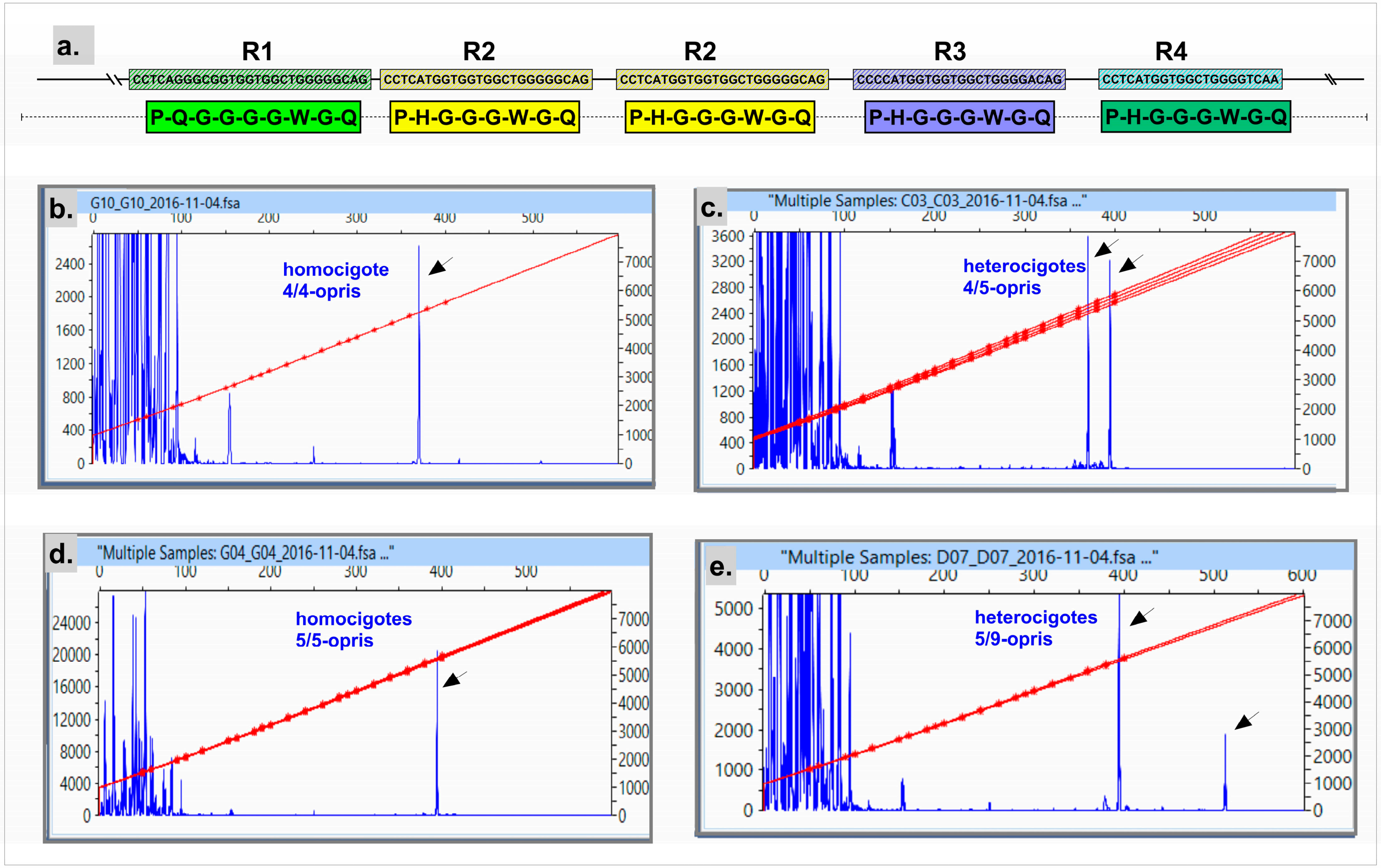

Figure S1. Fragment analysis for the *PRNP* gene. Panel a: Depicts the sequence regions (R1, R2, R3, R4) analyzed for *PRNP* gene fragments, indicating the specific sequences probed in the study. Panels b-e: Display capillary electrophoresis results for multiple samples, highlighting the detection of homozygotes and heterozygotes within the *PRNP* gene. Each panel corresponds to a different sample or condition: Panel b: Shows the fragment analysis for a homozygous sample labeled as "4/4-opris," with a peak indicating the presence of a specific homozygous state (HDL case). Panel c: Illustrates a heterozygous condition "4/5-opris" within the sample, with peaks corresponding to both homozygous and heterozygous states. Panel d: Like panel b, this depicts another homozygous sample "5/5-opris" with a clear peak for the homozygous state. Panel e: Presents fragment analysis for a sample from a Huntington's disease-like (HDL) condition, not Parkinson's disease, marked as "5/9-opris" heterozygotes, showing multiple peaks that indicate variability in the *PRNP* gene fragments. Each plot maps fluorescence intensity against fragment size (in base pairs) and includes a trend line (red) to indicate the expected size distribution of *PRNP* gene fragments.

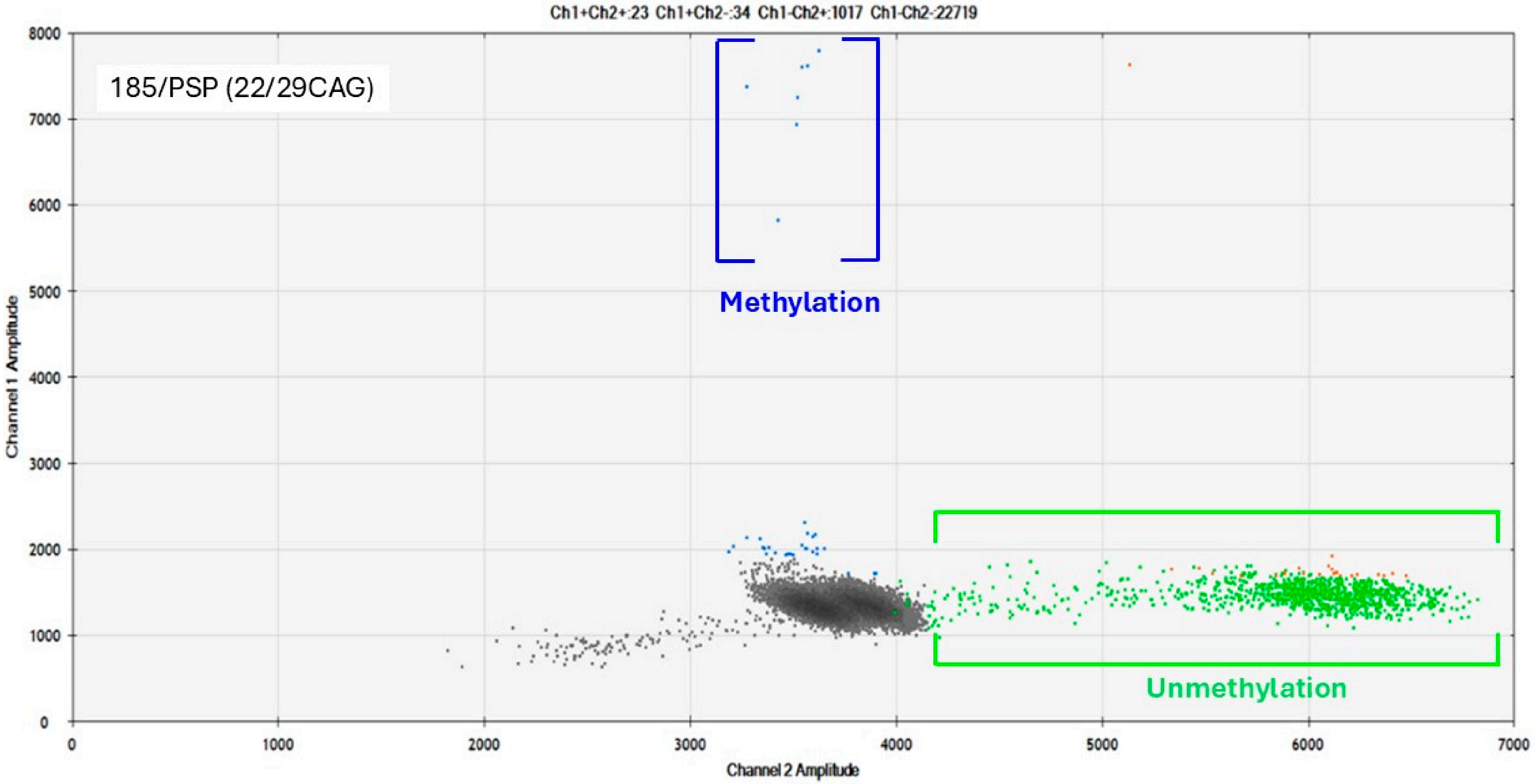

Figure S2. Digital PCR analysis of *ATXN2*-AS methylation in a PSP (Progressive Supranuclear Palsy) case with a genotype of 22/29 CAG repeats. The scatterplot depicts fluorescence amplitude on Channels 1 (VIC, vertical axis) and 2 (FAM, horizontal axis), categorizing droplet populations. Methylated droplets (blue rectangle): High signal in both channels, indicating methylated alleles. Unmethylated droplets (green rectangle): High signal in Channel 2 with low amplitude in Channel 1, representing unmethylated alleles. Negative droplets (black dots): Represent droplets lacking the target sequence, indicating no amplification. This analysis reveals distinct methylation and unmethylation states at the *ATXN2*-AS locus, contributing to the understanding of epigenetic regulation in PSP.

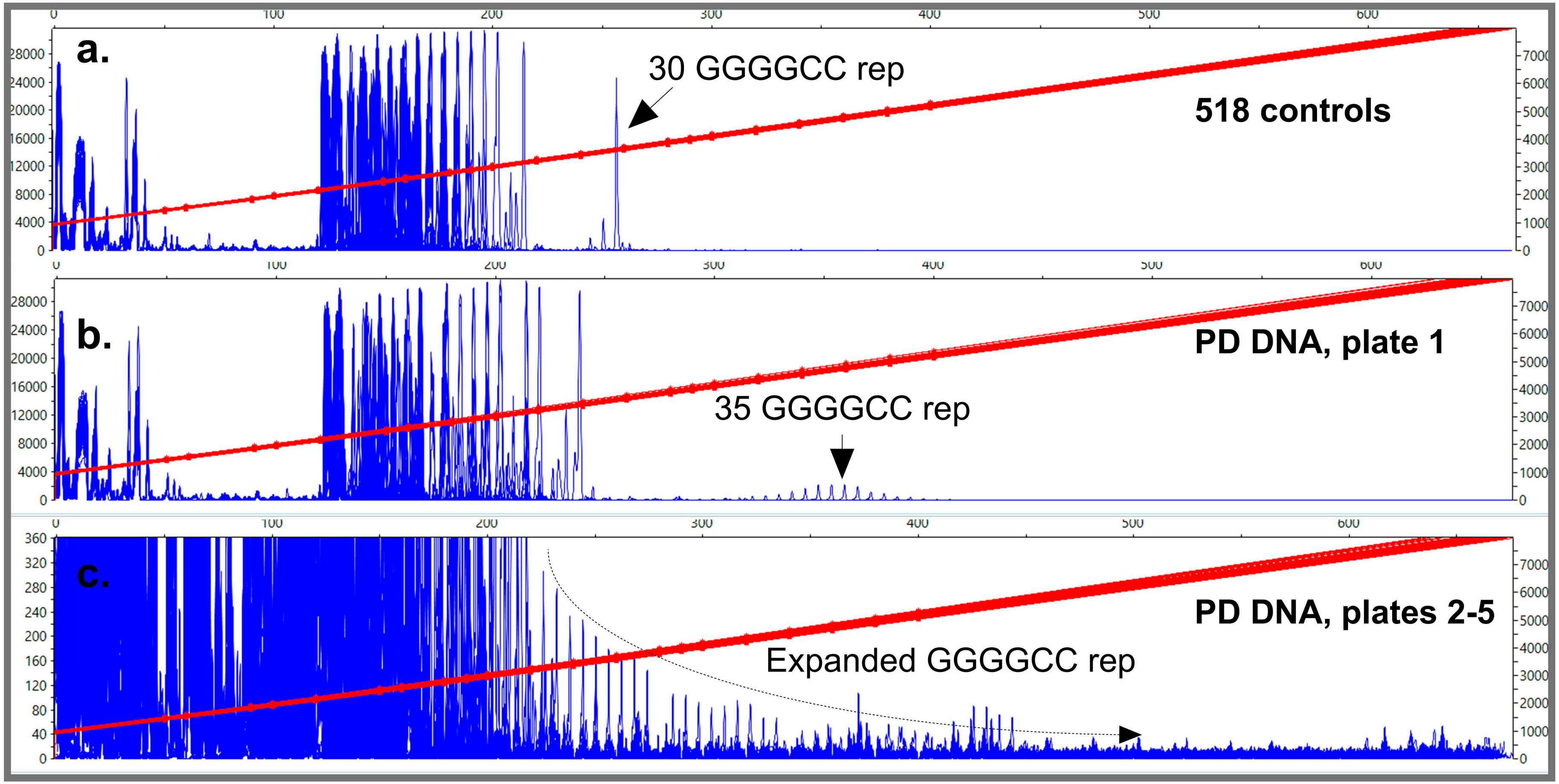

Figure S3. Electropherogram Profiles of *C9ORF72* GGGGCC Repeats in Control and PD Samples. (a) Control Samples. Representative electropherogram of C9ORF72 repeat analysis in 518 control samples. The arrow indicates the presence of a 30 GGGGCC repeat, consistent with an intermediate repeat range. (b) PD DNA Plate 1. Electropherogram showing a Parkinson's disease (PD) sample from plate 1. The arrow marks a 35 GGGGCC repeat, indicating a repeat length at the upper end of the normal range or potentially intermediate. (c) PD DNA Plates 2-5. Electropherogram of PD samples from plates 2–5. The profile demonstrates expanded GGGGCC repeats, as indicated by the broadened signal and dashed curve, consistent with pathological expansions observed in some PD cases.

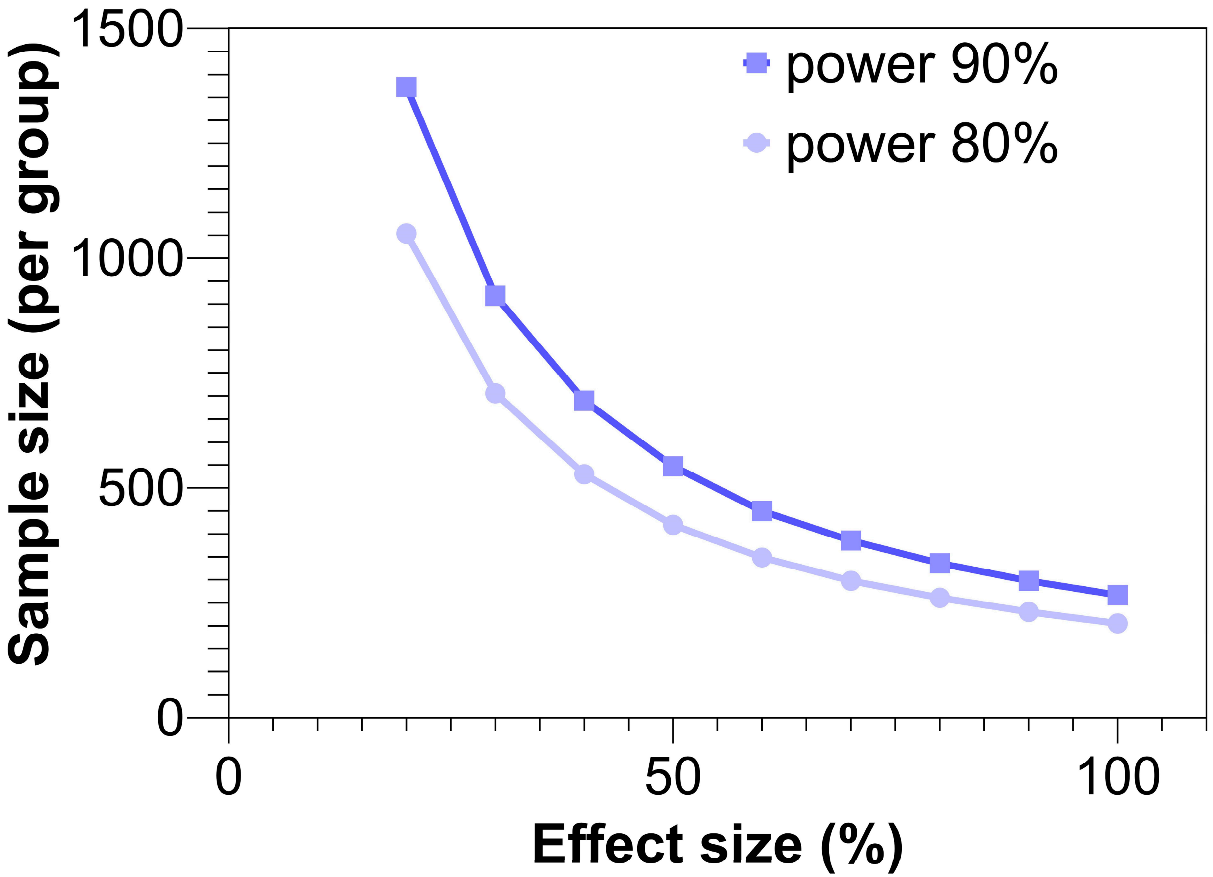

Figure S4. Power calculation to estimate the sample size required per group to detect an association of *TBP* long repeats with PD at varying effect sizes (x-axis). The plot illustrates the required sample sizes (y-axis) to achieve statistical power levels of 80% (light purple line) and 90% (dark purple line). As the effect size increases, the required sample size decreases, highlighting the relationship between effect size and statistical power in study design, group allocation 3.39 as used in this study. Using G*Power, we calculated the required sample sizes for a *priori* Fisher's exact test (Proportions: Inequality, two independent groups) across varying effect sizes (20%-100%) at power levels of 80% and 90%. The calculations were performed using the "A priori: Compute required sample size – given alpha, power, and effect size" function, with an alpha error set at 0.025 and an allocation ratio of 3.39.

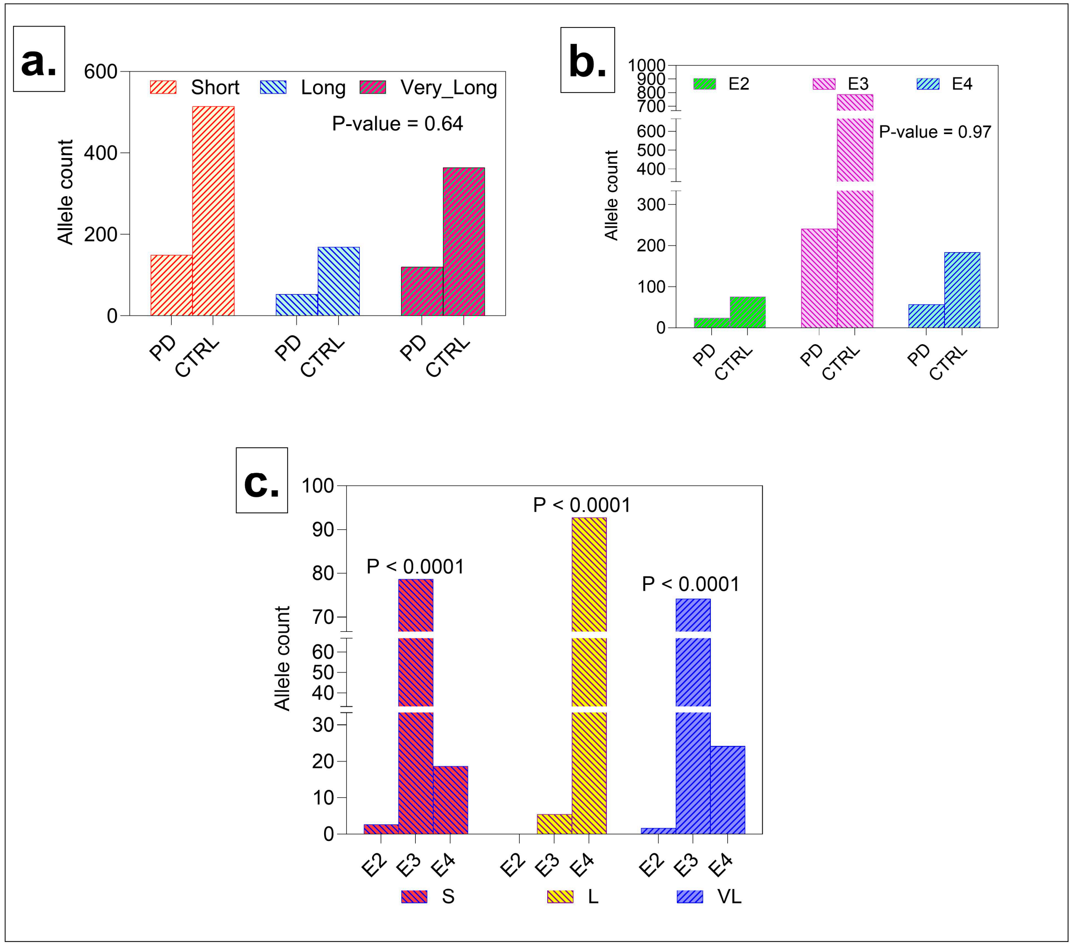

Figure S5. Distribution of APOE alleles and haplotypes in Parkinson's disease (PD) cases and controls. (a) Bar chart showing the frequency of APOE haplotypes grouped by repeat length categories (Short, Long, and Very Long) in PD cases and controls. No significant difference in the distribution of haplotype lengths is observed between the groups (P-value = 0.64). (b) Bar chart depicting the frequency of individual *APOE* alleles (E2, E3, and E4) in PD cases and controls. E3 is the most prevalent allele in both groups, followed by E4 and E2. No significant differences in allele distribution are noted (P-value = 0.97). (c) Association of APOE haplotypes (E2, E3, and E4) with *TOMM40* poly-T repeat length (Short (S), Long (L), and Very Long (VL)). Significant differences in the distribution of alleles within each length category are observed ($P < 0.0001$ for all comparisons), suggesting that haplotype length influences TOMM40 allele frequency distribution.

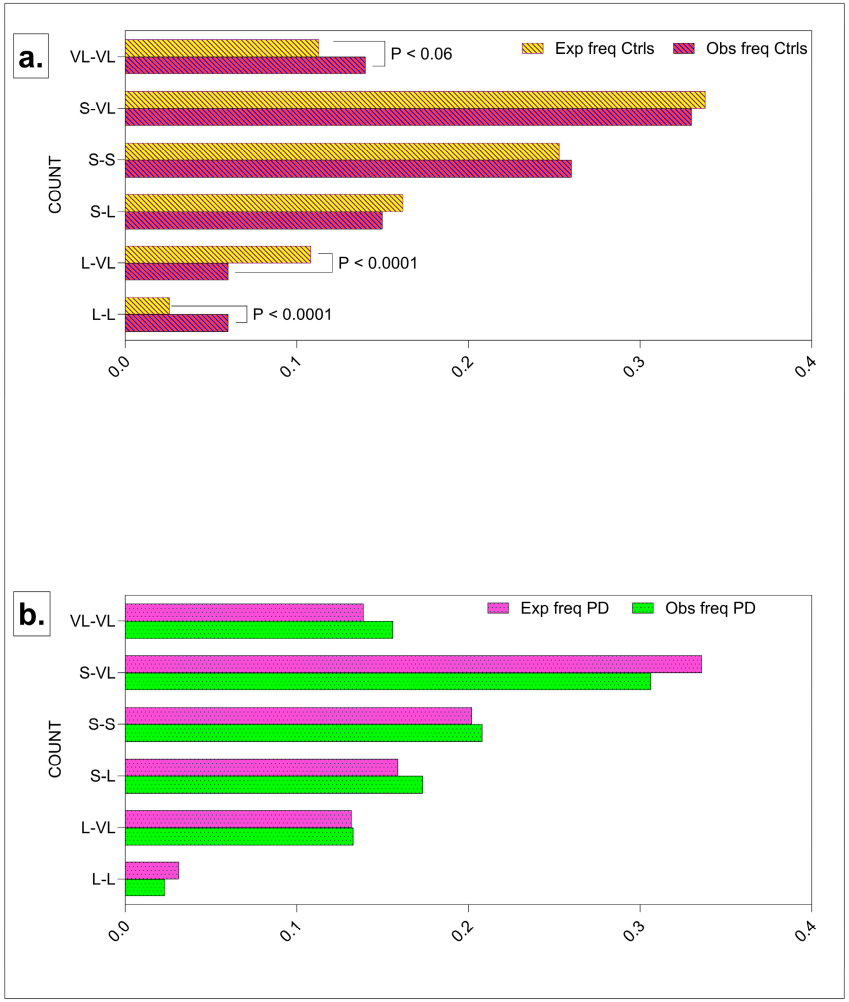

Figure S6. Hardy–Weinberg analysis for the *TOMM40* repeat length. Observed and expected frequencies of haplotypes in Parkinson's disease (PD) cases and controls. (a) Bar chart comparing the observed (solid bars) and expected (hatched bars) frequencies of different haplotype combinations (e.g., VL-VL, S-VL, etc.) in controls. Significant deviations from expected frequencies are indicated for specific haplotype pairs: L-VL and L-L combinations show significant differences (P < 0.0001). The VL-VL combination shows a trend toward significance (P < 0.06). (b) Bar chart illustrating the observed (solid bars) and expected (hatched bars) frequencies of haplotype combinations in PD cases.

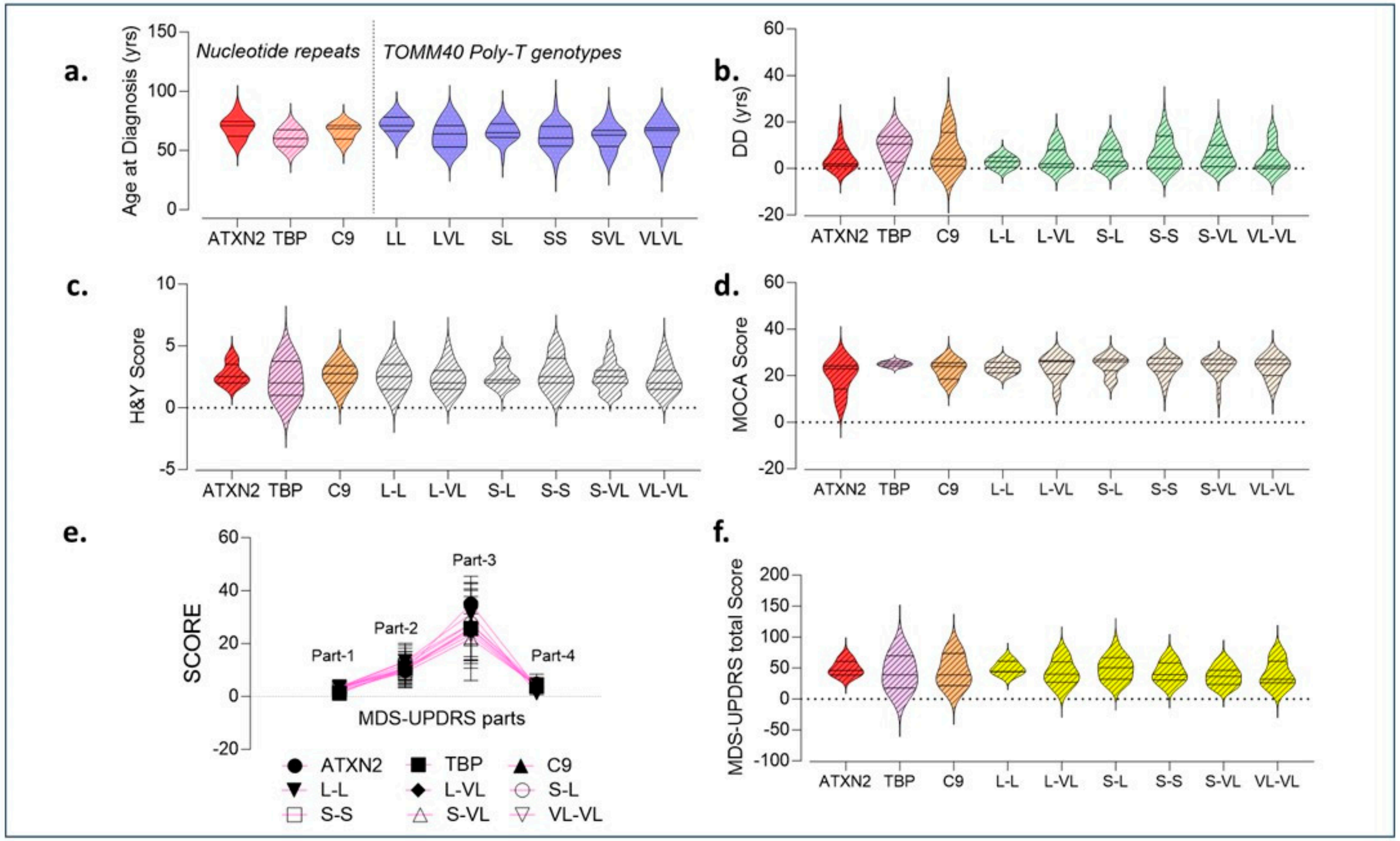

Figure S7. Clinical and genetic associations of nucleotide repeats and TOMM40 Poly-T genotypes with neurological scores. (a) Violin plots showing the distribution of age at diagnosis (years) for different nucleotide repeats (*ATXN2*, *TBP*, *C9ORF72*) and *TOMM40* Poly-T genotypes (LL, LVL, SL, SS, SVL, VL-VL). (b) Violin plots illustrating the disease duration (DD) (years) across the same genetic categories. No clear pattern of differentiation is observed for most genotypes. (c) Violin plots of the Hoehn and Yahr (H&Y) Score, indicating disease severity. The scores are largely consistent across genetic groups, with minimal variation between *TOMM40* Poly-T genotypes. (d) Violin plots displaying the MoCA (Montreal Cognitive Assessment) Score, a measure of cognitive function. (e) Line graph depicting the mean scores across MDS-UPDRS parts 1-4 (Movement Disorder Society-Unified Parkinson's Disease Rating Scale). (f) Violin plots for the MDS-UPDRS total score, combining parts 1-4.

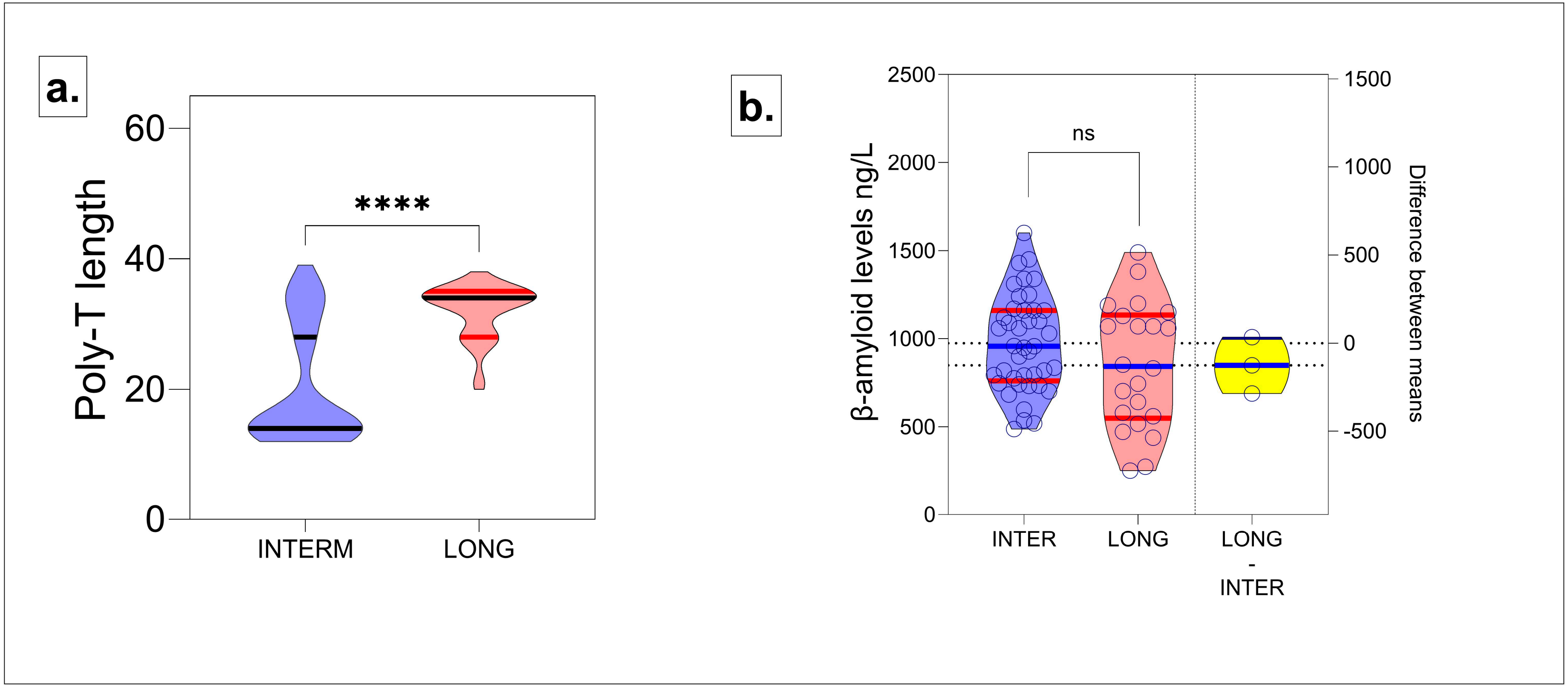

Figure S8. Poly-T Length and β-Amyloid Levels Across *TOMM40* Haplotypes. (a) Poly-T Length. Truncated violin plot showing a highly significant difference in Poly-T length between the INTERM and LONG haplotypes (****$p < 0.0001$). The INTERM haplotypes exhibit shorter Poly-T repeats compared to LONG haplotypes. (b) β-Amyloid Levels: Truncated violin plot showing no significant difference (ns) in β-amyloid levels between the INTERM and LONG haplotypes. The panel on the right illustrates the difference in means with a 95% confidence interval, confirming the lack of a statistically significant difference in β-amyloid levels between the two haplotype groups.

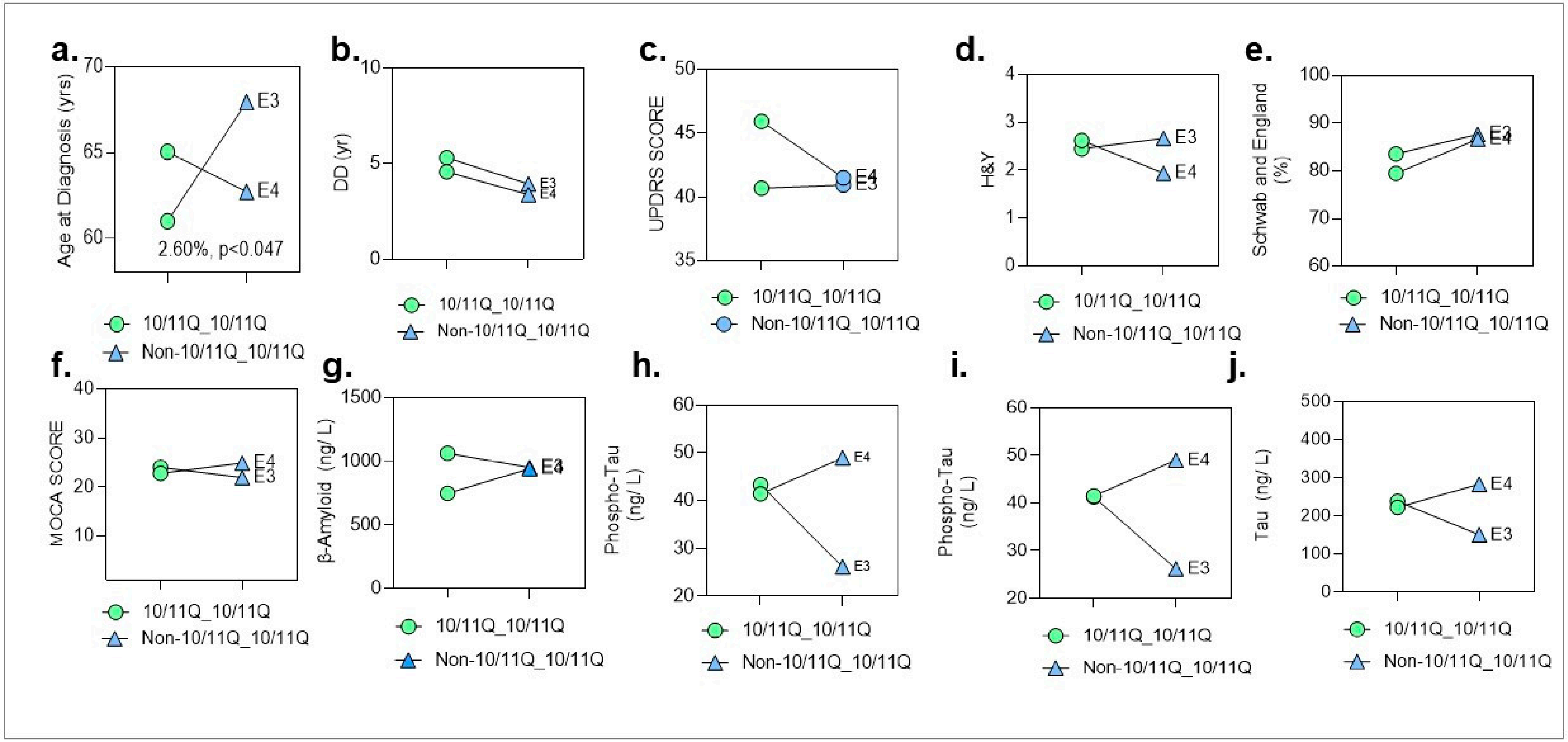

Figure S9. Interaction Plot resulting from the two-way ANOVA analysis *APOE* haplotype x *POLG* genotypes (10/11Q_10/11Q, Non-10/Non-11Q_10/11Q). Age at Diagnosis and Descriptive Analysis of Clinical and Biomarker Variables by APOE Genotype and *POLG* genotypes. This figure highlights the interaction between *APOE* genotypes (E3, E4) and 10/11Q vs. non-10/11Q groupings across various clinical and biomarker variables. (a) Age at Diagnosis: A significant difference was observed between APOE haplotypes (E3, E4) and *POLG* genotypes (10/11Q_10/11Q, Non-10/Non-11Q_10/11Q) (2.60%, $p < 0.047$). (b-j) For all other variables, including Disease Duration (b), UPDRS Score (c), Hoehn and Yahr (d), Schwab and England Scale (e), MoCA Score (f), β-Amyloid Levels (g), Phospho-Tau (h, i), and Total Tau (j), no statistically significant differences were detected.

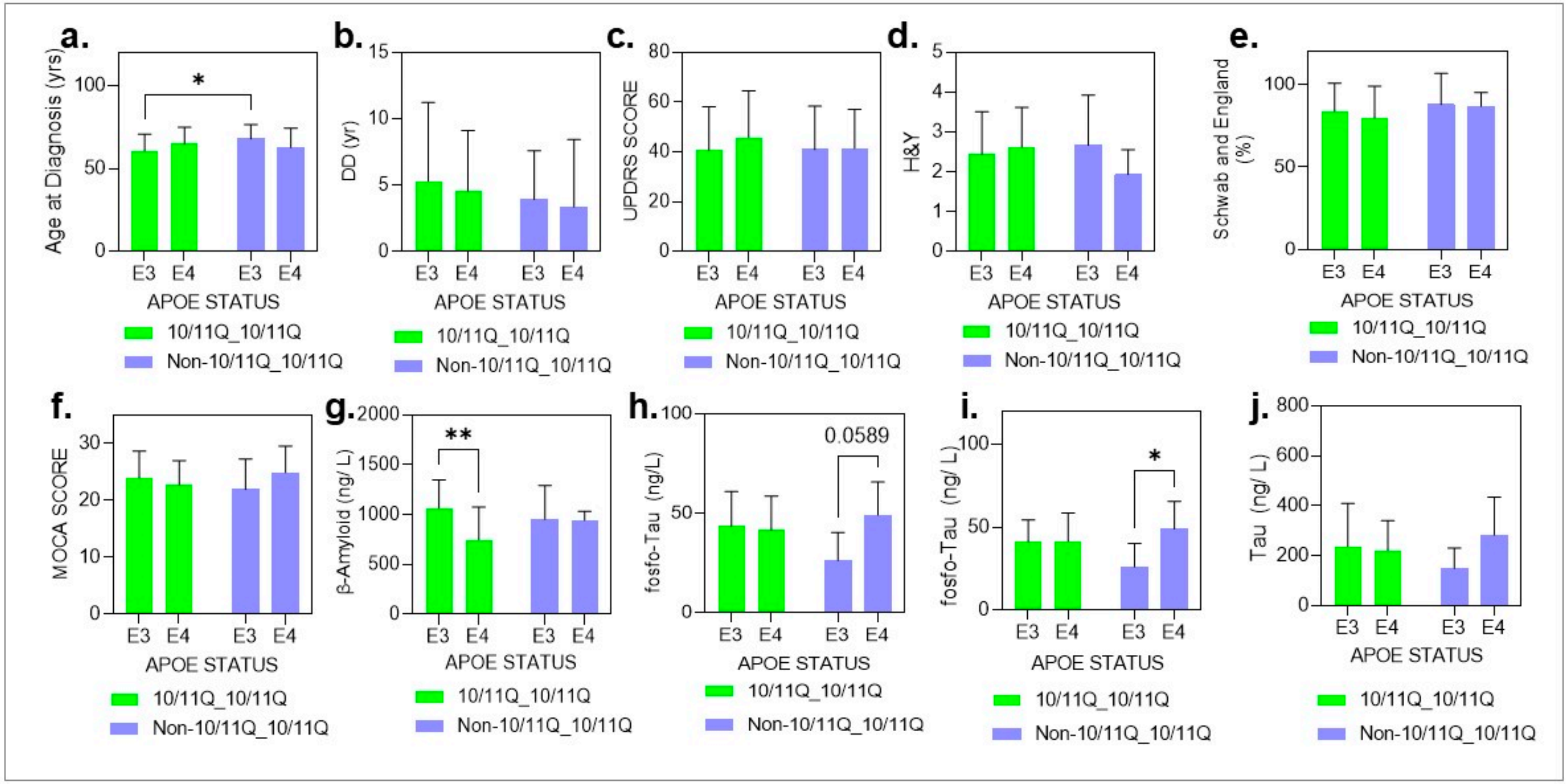

Figure S10. Clinical and Biomarker Profiles by APOE Genotype and *POLG* genotype grouping resulting in the two-way analysis *APOE* vs *POLG* genotypes. Comparison using ANOVA 2X2 of clinical and biomarker variables between APOE haplotypes (E3, E4) and groupings of POLG genotypes 10/11Q vs. Non-10/Non-11Q_10/11Q: (a) Age at Diagnosis: E3 carriers in the 10/11Q group had significantly earlier age at diagnosis compared to E3 carriers with Non-10/Non-11Q_10/11Q genotypes ($p < 0.05$). (b) Disease Duration (DD): No significant differences were observed between groups. (c) UPDRS Score: Similar scores were noted across all groups and genotypes. (d) Hoehn and Yahr (H&Y): No noticeable differences were detected between groups. (e) Schwab and England Scale: Scores remained consistent across groups and genotypes. (f) MoCA Score: Cognitive performance did not significantly differ across groups or genotypes. (g) β-Amyloid Levels: A significant reduction was observed in E4 carriers in the 10/11Q_10/11Q group (p < 0.01). (h) Phospho-Tau: Trends suggest higher levels in E3 carriers within the non-10/11Q group ($p = 0.0589$). (i) Phospho-Tau (ng/L) removing one outlier: Significantly higher levels were noted in E4 carriers within the Non-10/Non-11Q_10/11Q ($p < 0.05$). (j) Total Tau: No significant differences in total Tau levels were observed.

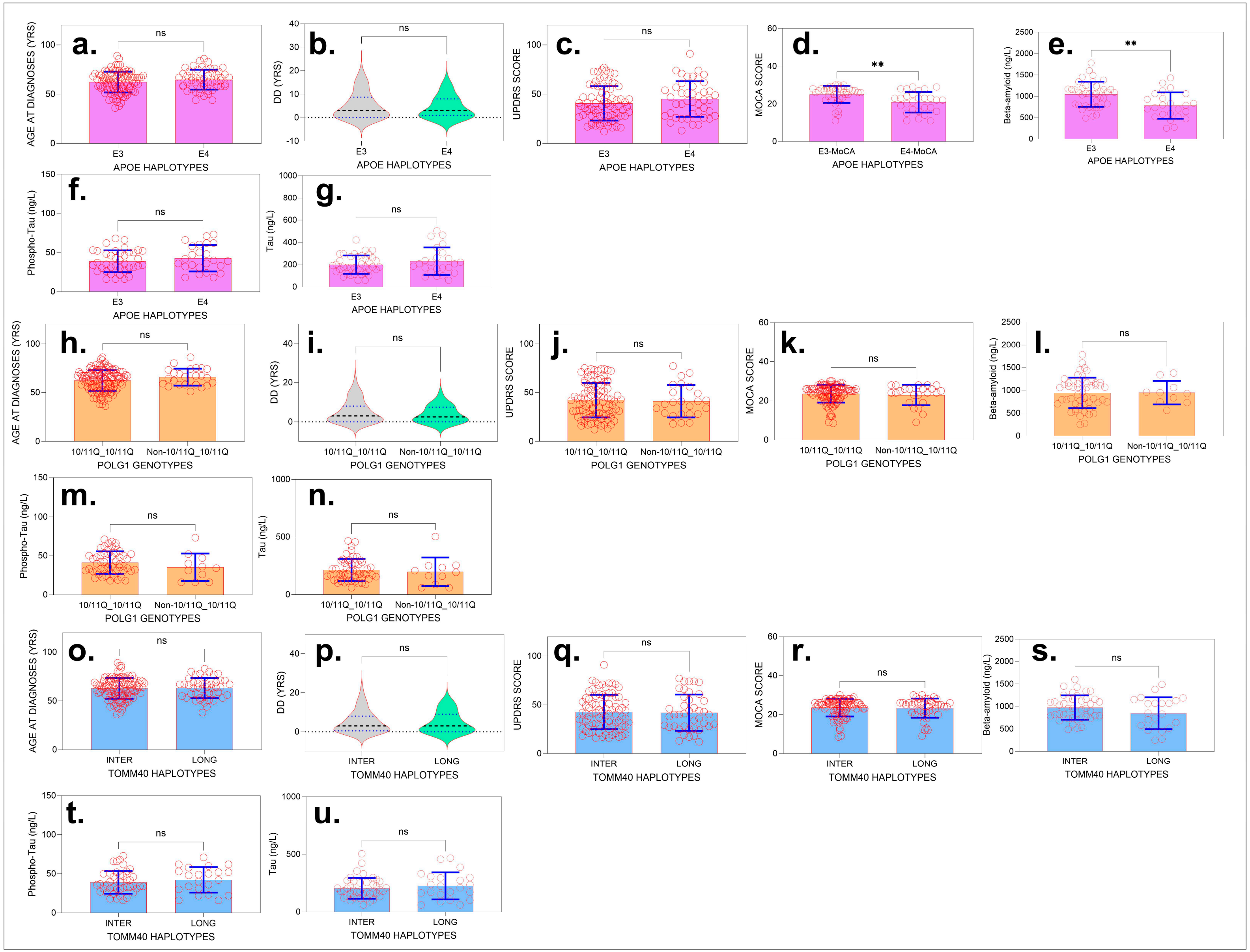
a.
b.
c.
d.
e.
f.
g.
h.
i.
j.
k.
l.
m.
n.
o.
p.
q.
r.
s.
t.
u.
ns
**
AGE AT DIAGNOSES (YRS)
DD (YRS)
UPDRS SCORE
MOCA SCORE
Beta-amyloid (ng/L)
Phospho-Tau (ng/L)
Tau (ng/L)
E3
E4
E3-MoCA
E4-MoCA
APOE HAPLOTYPES
10/11Q_10/11Q
Non-10/11Q_10/11Q
POLG1 GENOTYPES
INTER
LONG
TOMM40 HAPLOTYPES

Figure S11. Clinical and Biomarker Comparisons Across *APOE* Haplotypes, *POLG* Genotypes, and *TOMM40* Haplotypes. Comparison of clinical and biomarker measures stratified by *APOE* haplotypes, *POLG* genotypes, and *TOMM40* haplotypes: (a-e): *APOE* Haplotypes (a) Age at Diagnosis: No significant differences between haplotypes. (b) Disease Duration (DD): No significant differences. (c) UPDRS Score: No significant differences. (d) MoCA Score: Significant differences observed ($p < 0.01$) with reduced scores in E4 carriers. (e) β-Amyloid Levels: Significant increase in β-amyloid levels in E4 carriers ($p < 0.01$). (f-l): POLG Genotypes. (f) Phospho-Tau Levels: No significant differences. (g) Total Tau Levels: No significant differences. (h) Age at Diagnosis: No significant differences. (i) Disease Duration (DD): No significant differences. (j) UPDRS Score: No significant differences. (k) MoCA Score: No significant differences. (l) β-Amyloid Levels: No significant differences. (m-u): *TOMM40* Haplotypes. (m) Phospho-Tau Levels: No significant differences. (n) Total Tau Levels: No significant differences. (o) Age at Diagnosis: No significant differences. (p) Disease Duration (DD): No significant differences. (q) UPDRS Score: No significant differences. (r) MoCA Score: No significant differences. (s) β-Amyloid Levels: No significant differences. (t) Phospho-Tau Levels: No significant differences. (u) Total Tau Levels: No significant differences. Overall, the analysis reveals significant differences only for β-amyloid levels and MOCA scores within APOE haplotypes, particularly highlighting the impact of the E4 haplotype. Other genotype and haplotype comparisons showed no statistically significant differences across clinical and biomarker measures.

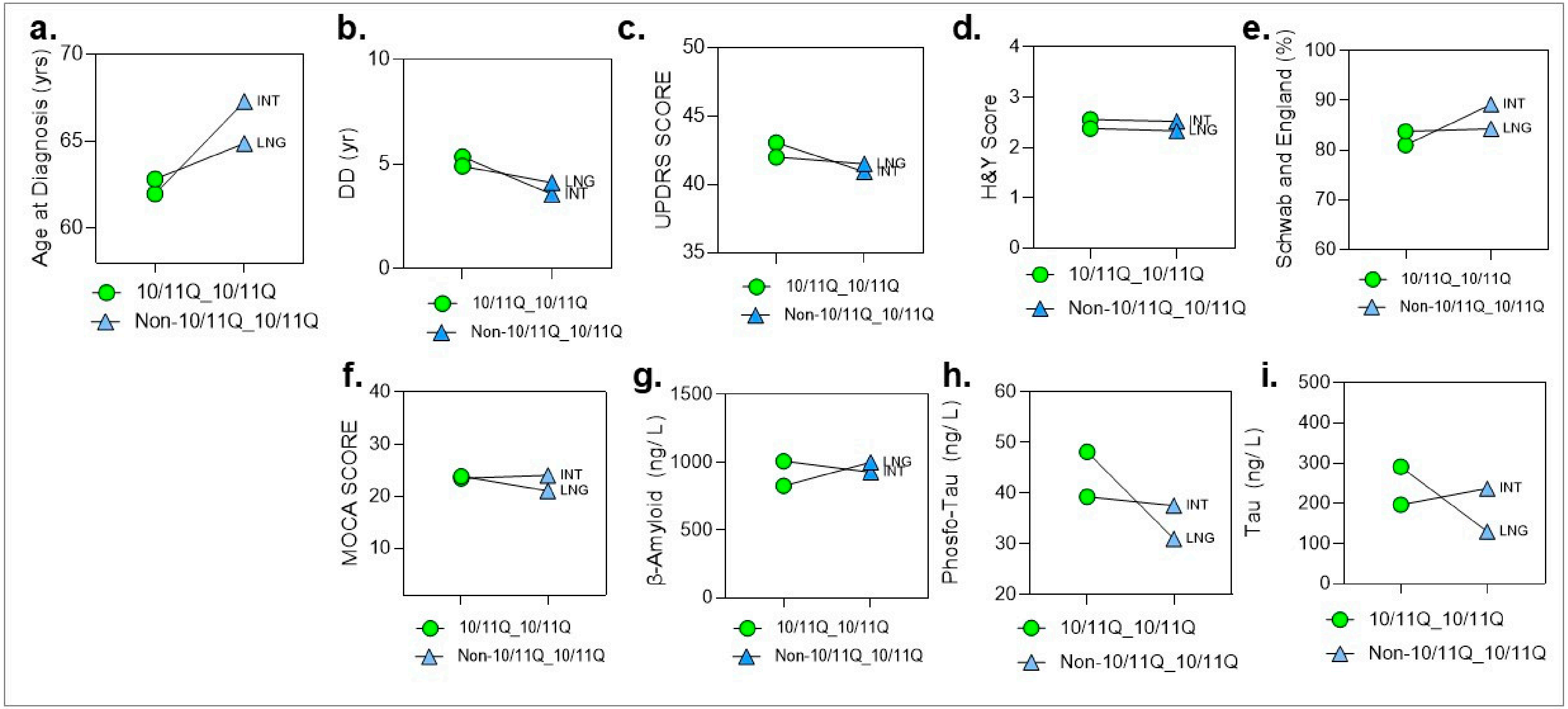

Figure S12. Interaction plot between *TOMM40* Genotype Length (INT vs. LNG) and *POLG* Status (10/11Q vs. Non-10/11Q) on Clinical and Biomarker Outcomes. (a-i): No significant differences or trends were observed across the assessed variables, including Age at Diagnosis (a), Disease Duration (b), UPDRS Score (c), Hoehn and Yahr Score (d), Schwab and England Scale (e), MoCA Score (f), β-Amyloid Levels (g), Phospho-Tau Levels (h), and (i) Total Tau Levels.

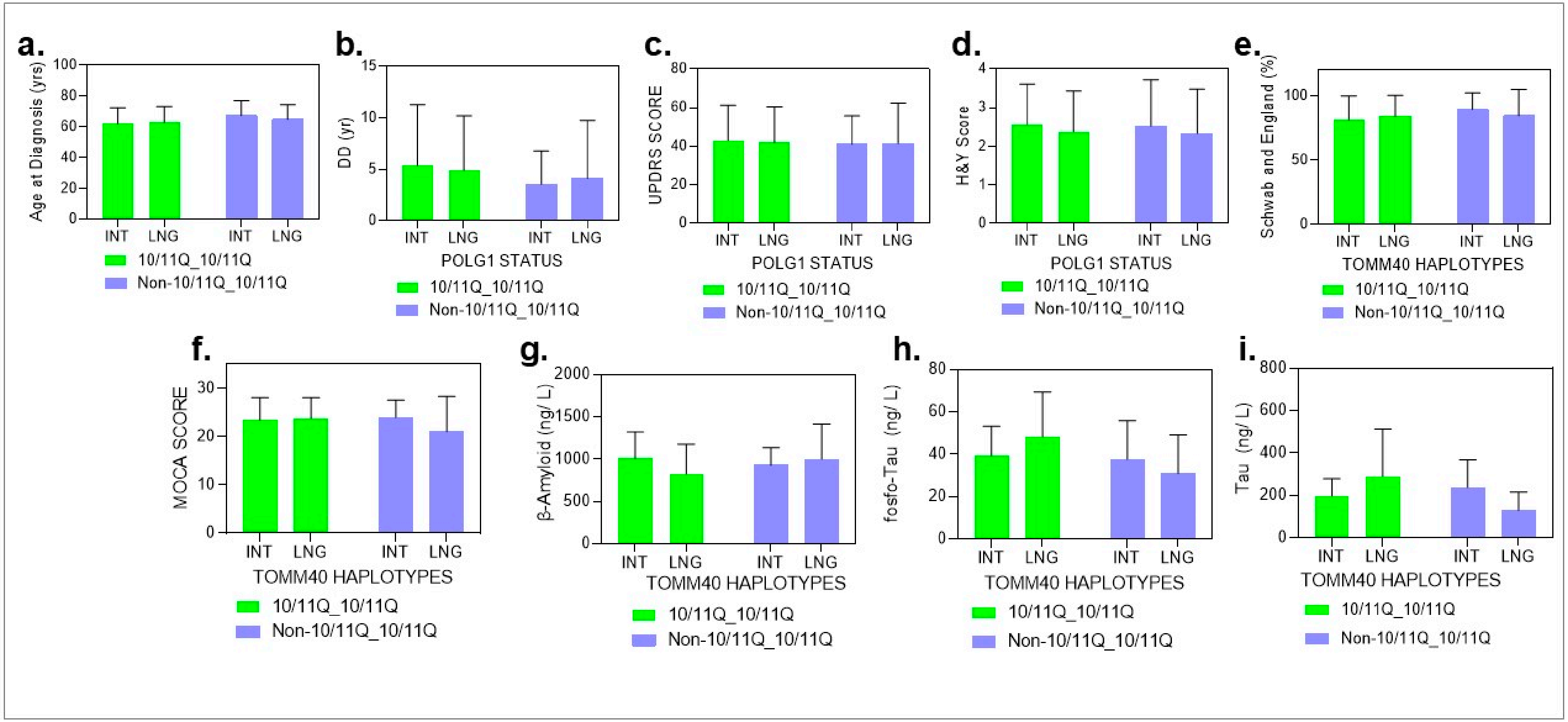

Figure S13. Analysis of *TOMM40* Haplotypes and *POLG* Status on Clinical and Biomarker Measures. (a) Age at Diagnosis: No significant differences are observed between groups or haplotypes. (b) Disease Duration (DD): No detectable differences are noted across the *POLG* and *TOMM40* categories. (c) UPDRS Score: Scores remain consistent between groups, showing no significant variations. (d) Hoehn and Yahr (H&Y) Score: No observable differences between groups or haplotypes. (e) Schwab and England Scale: Functional levels remain similar across all groupings and haplotypes. (f) MoCA Score: Cognitive performance is consistent across both haplotypes and *POLG* genotypes. (g) β-Amyloid Levels: Levels are similar between groups, showing no significant trends or differences. (h) Phospho-Tau Levels: No significant variation is observed across haplotypes or *POLG* groups. (i) Total Tau Levels: Tau concentrations are comparable across all groups and haplotypes.

P-values	ADx	REPEATS	APOE E4	TOMM40	POLG1	MALE?	DD	Heredity	UPDRS	H&Y	MADRS	MoCA	β-Amyloid	fosfo-Tau	TAU
ADx		0,31	0,16	0,80	0,05	0,03	0,00	0,17	0,00	0,01	0,81	0,01	0,15	0,057	0,009
REPEATS	0,31		0,57	0,88	0,49	0,22	0,87	0,74	0,85	0,32	0,30	0,06	0,75	0,891	0,981
APOE E4	0,16	0,57		0,00	0,47	0,91	0,37	0,59	0,25	0,90	0,53	0,57	0,00	0,591	0,766
TOMM40	0,80	0,88	0,00		0,91	0,63	0,77	0,09	0,83	0,35	0,64	0,86	0,09	0,170	0,122
POLG1	0,05	0,49	0,47	0,91		0,13	0,23	0,18	0,70	0,88	0,33	0,62	0,96	0,209	0,517
MALE?	0,03	0,22	0,91	0,63	0,13		0,85	0,07	0,31	0,14	0,50	0,20	0,76	0,401	0,934
DD	0,00	0,87	0,37	0,77	0,23	0,85		0,06	0,01	0,00	0,72	0,82	0,62	0,636	0,410
Heredity	0,17	0,74	0,59	0,09	0,18	0,07	0,06		0,35	0,02	0,59	0,03	0,74	0,687	0,838
UPDRS	0,00	0,85	0,25	0,83	0,70	0,31	0,01	0,35		0,00	0,00	0,00	0,01	0,845	0,297
H&Y	0,01	0,32	0,90	0,35	0,88	0,14	0,00	0,02	0,00		0,00	0,00	0,02	0,151	0,082
MADRS	0,81	0,30	0,53	0,64	0,33	0,50	0,72	0,59	0,00	0,00		0,03	0,13	0,327	0,032
MoCA	0,01	0,06	0,57	0,86	0,62	0,20	0,82	0,03	0,00	0,00	0,03		0,00	0,571	0,472
β-Amyloid	0,15	0,75	0,00	0,09	0,96	0,76	0,62	0,74	0,01	0,02	0,13	0,00		0,003	0,085
fosfo-Tau	0,06	0,89	0,59	0,17	0,21	0,40	0,64	0,69	0,85	0,15	0,33	0,57	0,00		0,000
TAU	0,01	0,98	0,77	0,12	0,52	0,93	0,41	0,84	0,30	0,08	0,03	0,47	0,08	0,000	

	ADx	REPEATS	APOE E4	TOMM40	POLG1	MALE?	DD	Heredity	UPDRS	H&Y	MADRS	MoCA	β-Amyloid	fosfo-Tau	TAU
ADx	152	152	152	152	152	152	122	151	118	148	111	125	64	65	65
REPEATS	152	159	159	159	159	159	124	158	121	154	113	128	64	65	65
APOE E4	152	159	159	159	159	159	124	158	121	154	113	128	64	65	65
TOMM40	152	159	159	159	159	159	124	158	121	154	113	128	64	65	65
POLG1	152	159	159	159	159	159	124	158	121	154	113	128	64	65	65
MALE?	152	159	159	159	159	159	124	158	121	154	113	128	64	65	65
DD	122	124	124	124	124	124	124	124	119	123	106	112	57	58	58
Heredity	151	158	158	158	158	158	124	158	121	153	113	128	64	65	65
UPDRS	118	121	121	121	121	121	119	121	121	120	105	111	56	57	57
H&Y	148	154	154	154	154	154	123	153	120	154	110	126	63	64	64
MADRS	111	113	113	113	113	113	106	113	105	110	113	103	52	53	53
MoCA	125	128	128	128	128	128	112	128	111	126	103	128	59	60	60
β-Amyloid	64	64	64	64	64	64	57	64	56	63	52	59	64	64	64
fosfo-Tau	65	65	65	65	65	65	58	65	57	64	53	60	64	65	65
TAU	65	65	65	65	65	65	58	65	57	64	53	60	64	65	65

Figure S14. Multiple Linear Regression Analysis. P-Values, Parameters, and Sample Sizes Highlighting Relationships Between Key Clinical, Genetic, and Biomarker Variables in the Cohort. The top section displays P-values, with significant values ($P < 0.05$) highlighted in red and non-significant values in blue, assessing correlations between factors like gene repeats, *APOE E4, TOMM40, POLG* status, and clinical outcomes such as UPDRS, MADRS, MoCA, and biomarkers like β-Amyloid and Tau. The bottom section shows the sample sizes for each analysis. This visualization aids in identifying key relationships and variances within the data set.

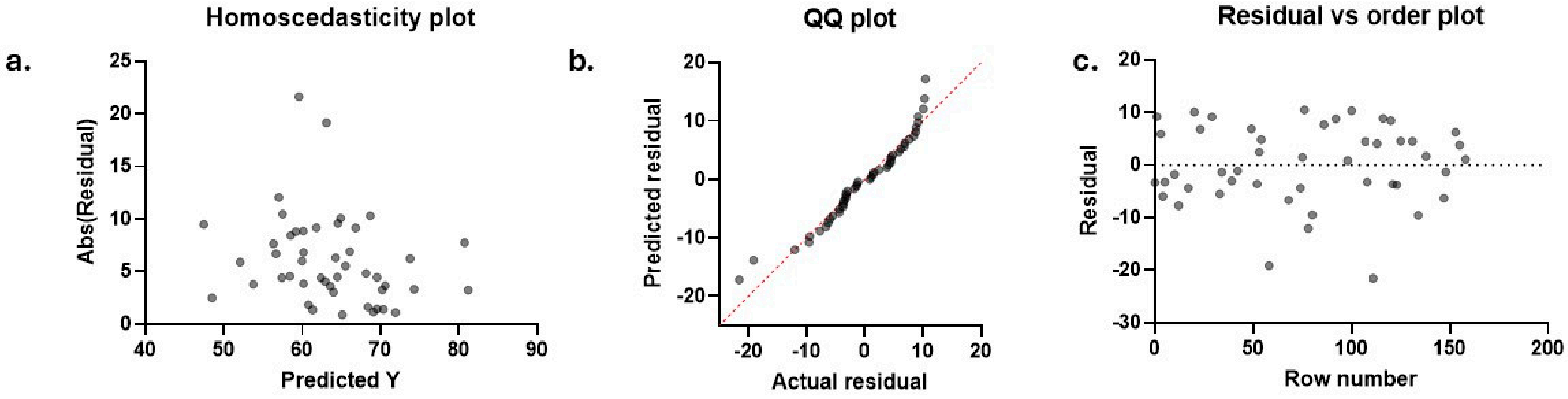

Figure S15. QC parameters for Multiple Linear Regression Analysis Between Key Clinical, Genetic, and Biomarker Variables in the PD Cohort. (a) Homoscedasticity Plot - Displays the absolute residuals against the predicted values (Predicted Y) to assess the constancy of variance across the range of predictions. A random spread of points suggests appropriate homoscedasticity. (b) Quantile-Quantile (QQ) Plot - Compares the distribution of the predicted residuals against a theoretical normal distribution (represented by the red dashed line). Deviations from the line indicate deviations from normality in the residual distribution. (c) Residual vs. Order Plot - Shows the residuals plotted against the row number of the data set to check for any patterns that might suggest non-randomness or time-related trends in the residuals. The absence of a clear pattern supports the assumption of independence among residuals.

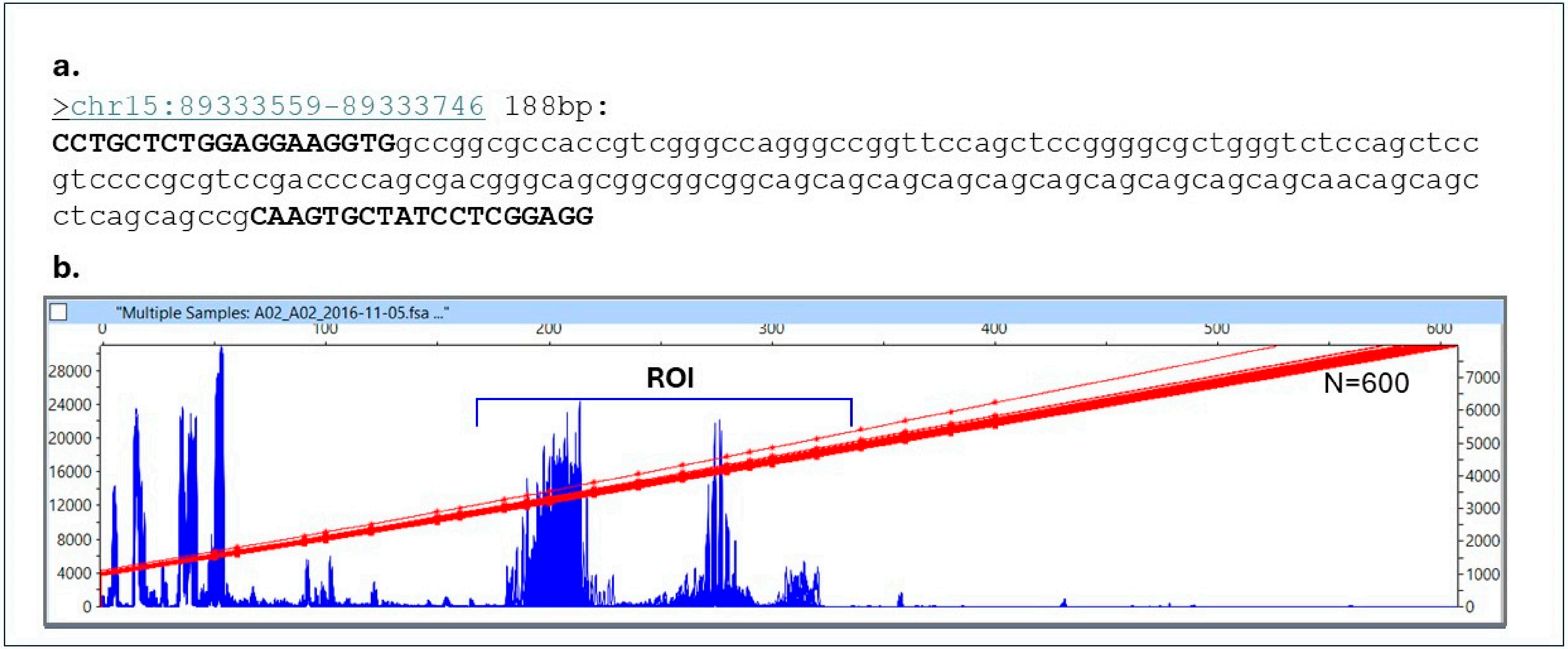

Figure S16. Amplicon sequence and fragment analysis data for the *POLG* gene from a PD cohort. (a) Shows the sequence of the expected 188 bp amplicon from the POLG gene, located on chromosome 15, highlighting the specific genomic region (89333559–89333746). The sequence includes relevant nucleotide details that are essential for subsequent analyses. (b) Displays an overlay of capillary electrophoresis fragment analysis for multiple samples within the PD cohort, with a focus on the Region of Interest (ROI). The graph plots fluorescence intensity against the amplicon size (in base pairs), demonstrating the distribution of fragment sizes within the cohort. The ROI box highlights the expected location of the *POLG* amplicon, correlating the observed data with the expected sequence characteristics. The trend line (red) indicates the general distribution of detected fragments, and the total number of analyzed fragments (N=600) is noted, providing a statistical basis for evaluating *POLG* variation in the PD cohort.

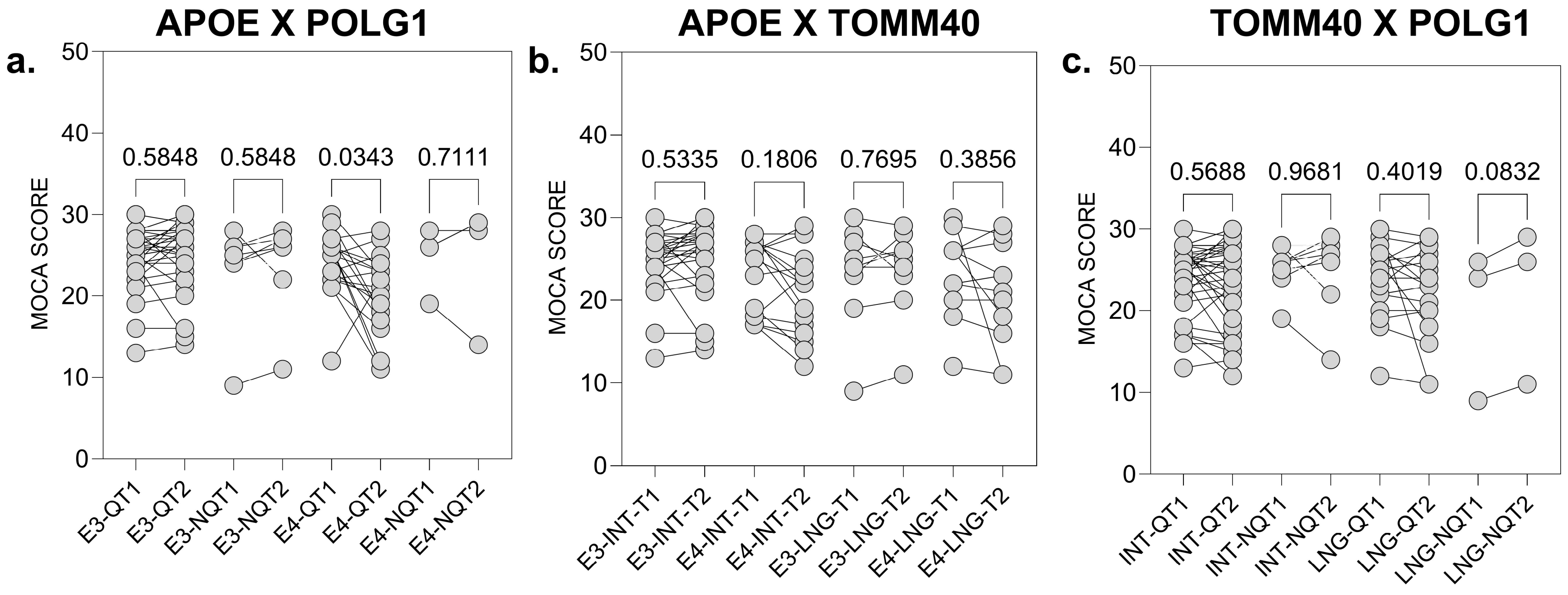

Figure S17. Interaction effects of haplotype combinations from the *APOE, TOMM40*, and *POLG* genes on the MoCA scores over two time points, T1 and T2. The panels (a-c) display individual participant scores, allowing for direct comparison of the changes in cognitive performance associated with different genetic interactions across time. a) Displays MoCA scores for interactions between APOE and POLG haplotypes (E3-Q, E3-NQ, E4-Q, E4-NQ, Q=10Q, NQ= N10/11Q) at both T1 and T2. Q-values above the comparisons assess the significance, using FDR BKY, of changes across time points. b) Illustrates the impact of *APOE* and *TOMM40* haplotype interactions (E3-INT1, E4-INT1, E3-LNG, E4-LNG) on MOCA scores. The variations between T1 and T2 are visualized with associated p-values indicating the level of statistical significance. c) Focuses on TOMM40 (INT and LNG) and POLG (Q and NQ, Q=10Q, NQ= N10/11Q) haplotype interactions affecting MoCA scores over the two time points. Each panel uses connecting lines to depict the change in scores for individual participants, providing a clear visual representation of the extent and direction of changes over time. The q-values positioned above each plot inform on the statistical relevance of these observations, aiding in the interpretation of how these genetic interactions influence cognitive functions longitudinally.

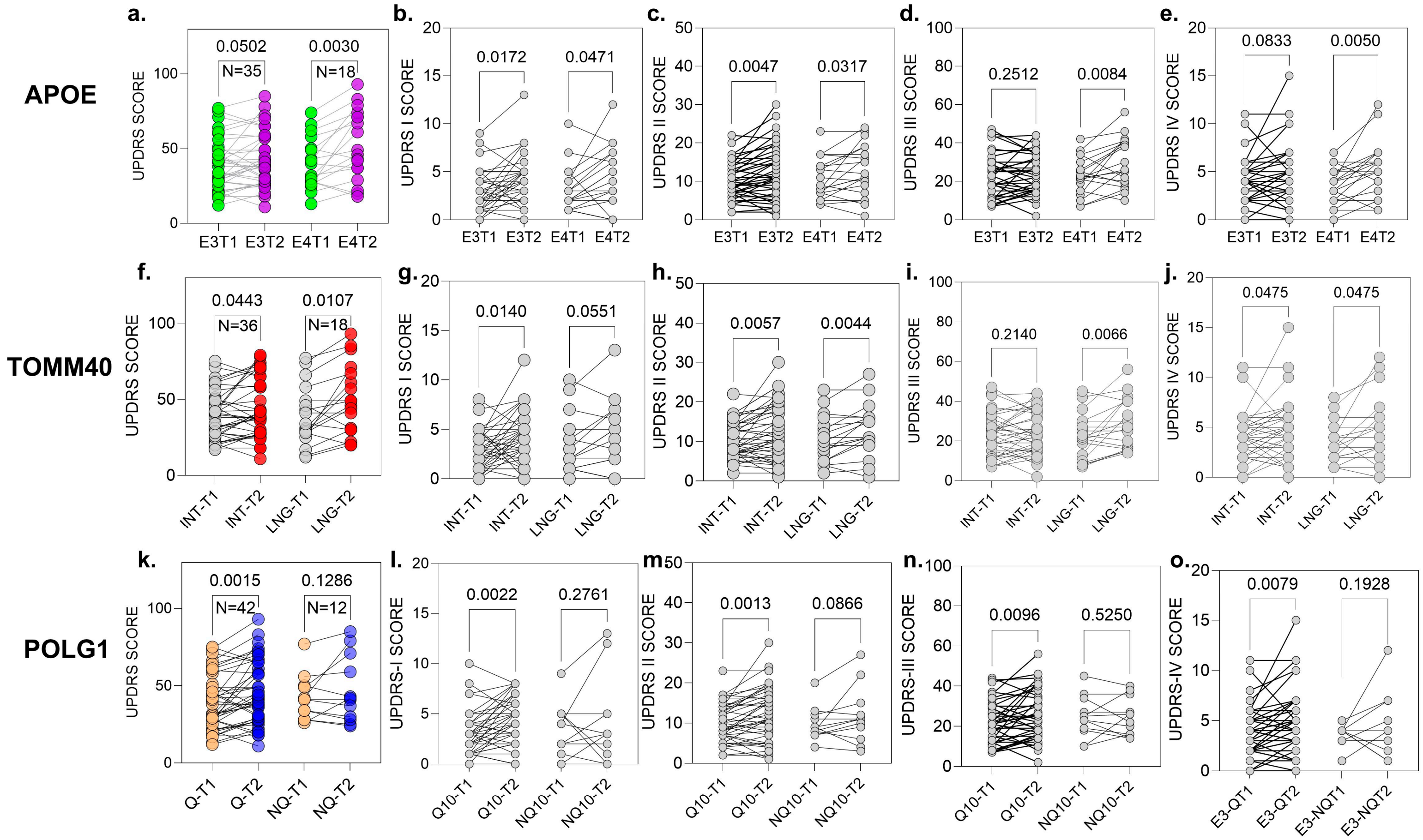

Figure S18. Longitudinal analyses of UPDRS scores associated with specific haplotypes within the *APOE*, *TOMM40*, and *POLG* genes over two time points, T1 and T2. The panels (a-o) depict before-and-after plots, showing individual participant scores to demonstrate changes over time within each haplotype group. Panels a-e focus on APOE haplotypes and times T1 T2, E3-T1, E3-T2, E4-T1, and E4-T2, illustrating the variability in UPDRS scores within these haplotype groups between T1 and T2. Panels f-j present data for TOMM40 haplotypes INT-T1, IN-T2, LNG-T1, and LNG-T2, highlighting differences in motor scores across the two time points. Panels k-o show the scores for *POLG* haplotypes Q-T1, Q-T2, NQ-1T1, and NQ-T2, similarly tracking the changes in UPDRS scores over time, (Q=10Q, NQ= N10/11Q). The q-values displayed above each plot provide statistical insight into the changes observed, with significance indicated for comparisons where q < 0.05.

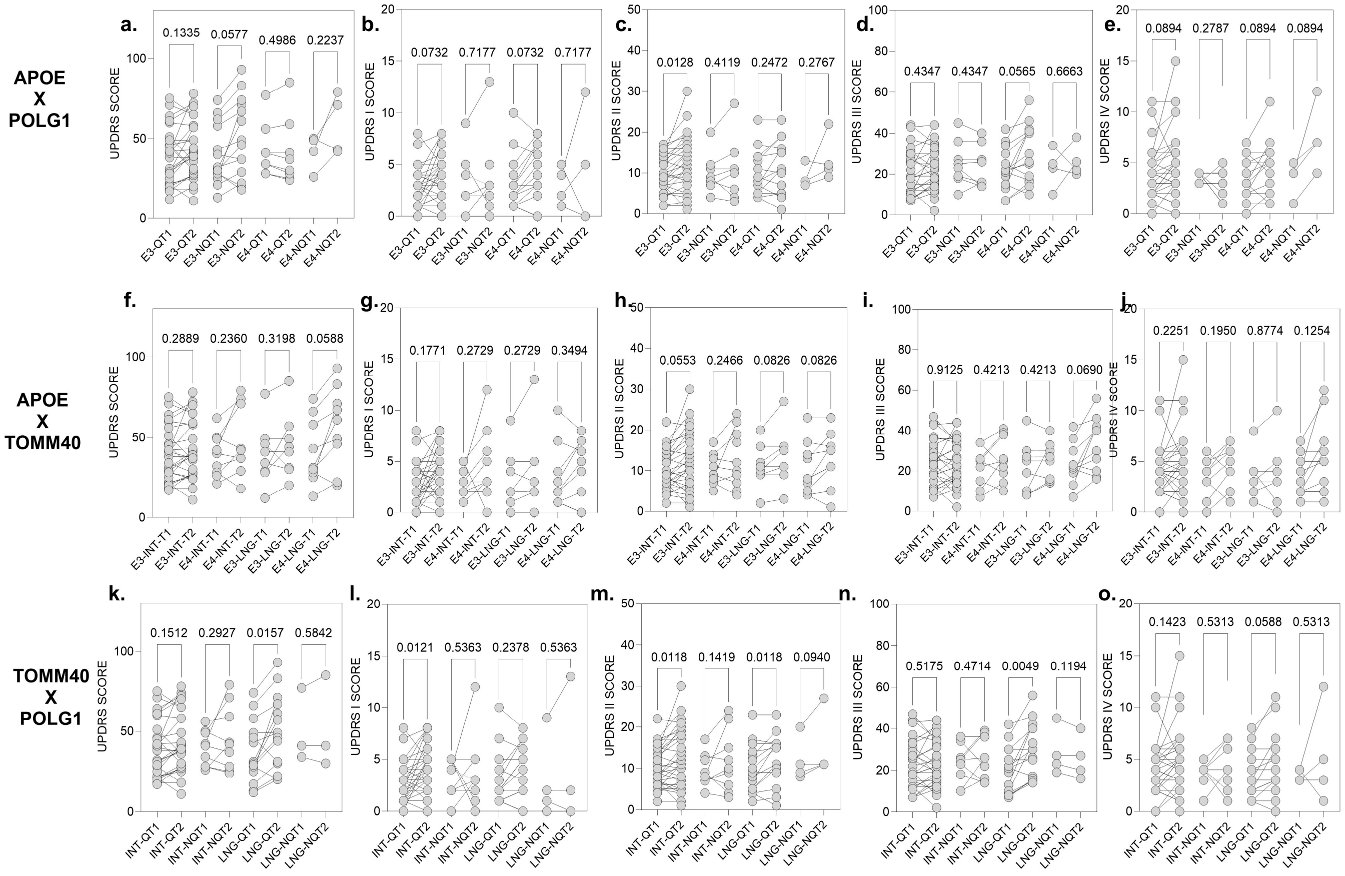

Figure S19. Interaction effects between haplotypes of APOE, TOMM40, and POLG genes on UPDRS scores at two time points, T1 and T2. The analyses are grouped into three main interactions across multiple panels: APOE x POLG (Panels a-e), TOMM40 x POLG (Panels f-j), and APOE x TOMM40 (Panels k-o). Panels a-e: Explore the interactions between APOE haplotypes (E3, E4) and POLG haplotypes (Q, NQ, Q=10Q, NQ= N10/11Q) and their impact on UPDRS scores, showing individual participant changes from T1 to T2. Panels f-j: Assess the interactions between APOE haplotypes (E3, E4) and TOMM40 haplotypes (INT, LNG), illustrating changes in UPDRS scores. Panels k-o: Focus on the interactions between TOMM40 haplotypes (INT, LNG) and POLG haplotypes (Q, NQ, Q=10Q, NQ= N10/11Q), detailing the associated UPDRS score variations across the two time points. Each panel displays longitudinal data with individual changes highlighted by connecting lines, providing visual evidence of haplotype impact on disease progression as measured by UPDRS. Q-values, listed above each plot, indicate the statistical significance of changes observed.

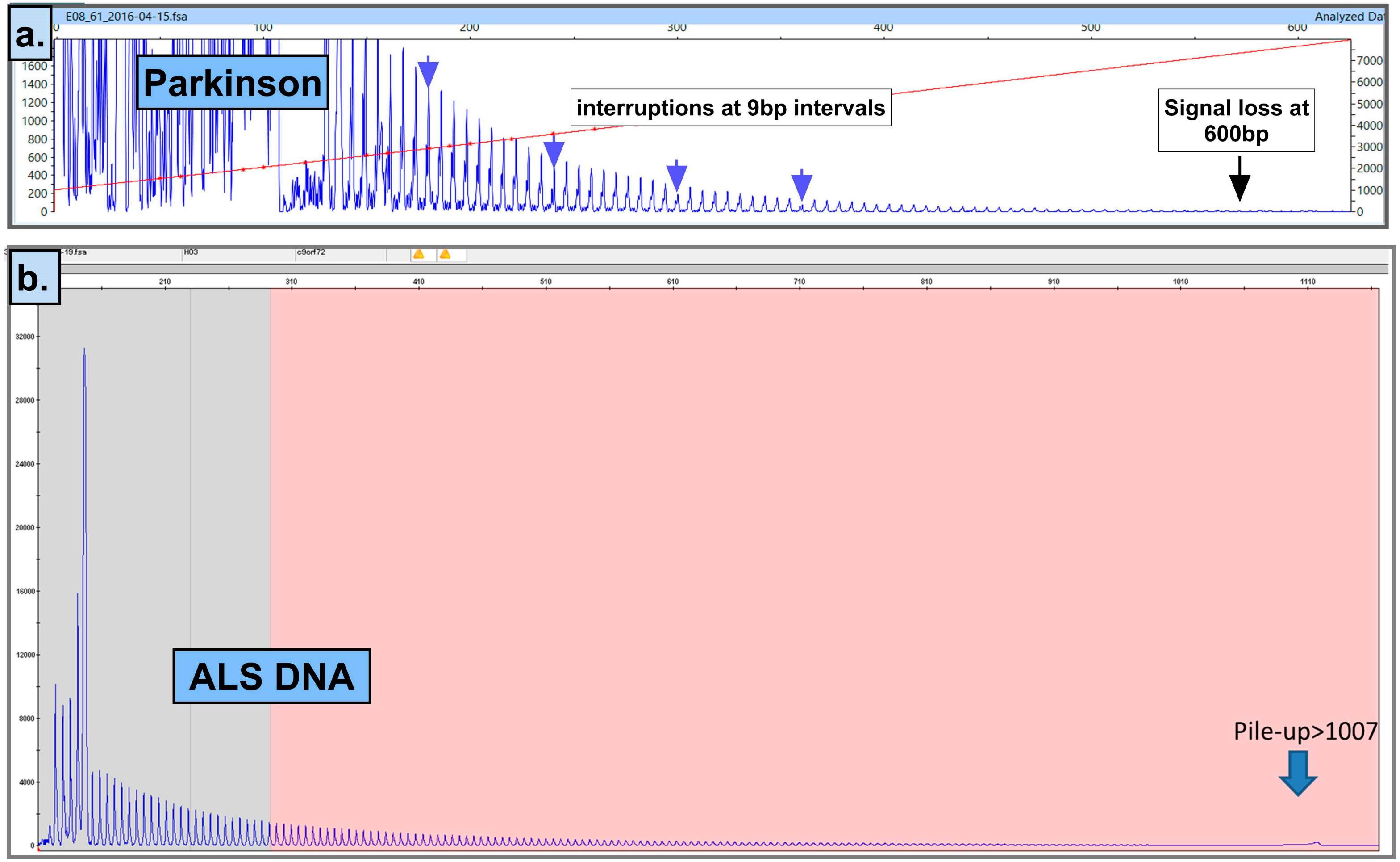

Figure S20. Comparison of typical fragment length analysis profiles for PD (Panel a) and ALS (Panel b). Panel a (Parkinson): The electropherogram shows DNA fragments with interruptions occurring at 9 bp intervals, and a notable signal loss at approximately 600 bp, indicative of moderate fragment length sizes. Panel b (ALS): The electropherogram highlights a pile-up of fragments exceeding 1007 bp, characteristic of ALS DNA, with a distinct accumulation in the higher fragment size range. This analysis demonstrates the differences in DNA fragment patterns between the two neurodegenerative diseases, reflecting their distinct genetic profiles.

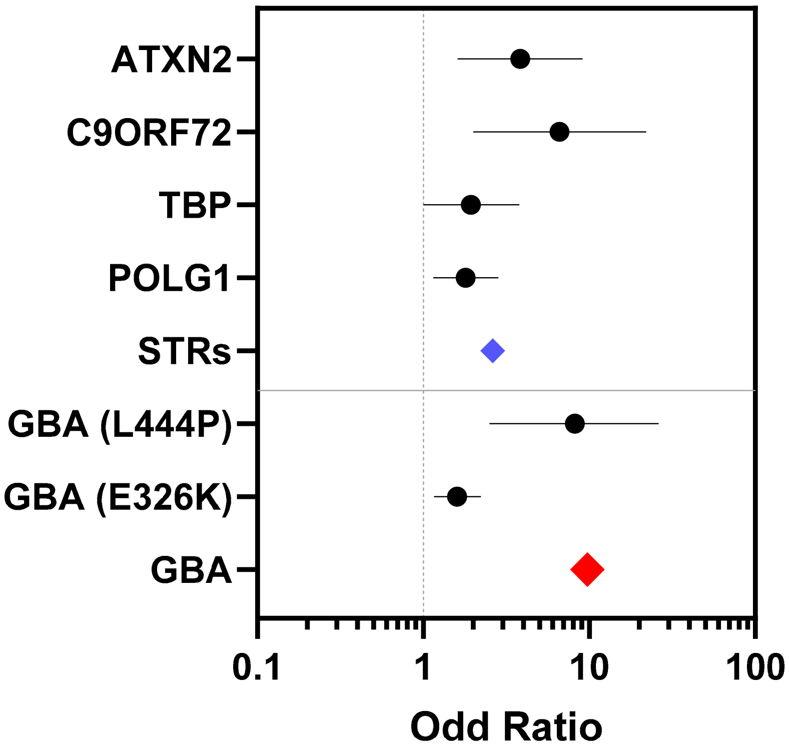

Figure S21. Genetic association analysis of variants linked to Parkinson's disease risk in a Swedish cohort. Odds ratios (OR) are plotted on a logarithmic scale for pathogenic or risk-associated variants in genes implicated in neurodegeneration (*ATXN2*, *C9ORF72*, *TBP*, *POLG*, STRs, and *GBA*). Black circles represent point estimates of ORs, with horizontal lines indicating 95% confidence intervals. The blue diamond corresponds to a repeat expansion marker (STR), while the red diamond reflects the combined effect of all *GBA* variants analyzed. The vertical dashed line at OR = 1 denotes the null hypothesis of no association. ORs greater than 1 suggest increased disease risk.
